
JOURNAL INFORMATION
==============================
NLM Title Abbreviation: Immun Inflamm Dis
Journal ID: Immun Inflamm Dis
NLM Journal ID: 101635460
Journal ID: IID3

Immunity, Inflammation and Disease

EISSN: 2050-4527
Publisher: Wiley

ARTICLE INFORMATION
==============================
PMCID: PMC13548399
DOI: 10.1002/iid3.70525
Article ID: IID370525
Article version: 1
Subjects: Issue Information, Issue Information

Issue Information

Electronic publication date: 2026 Sep 7
Publication date: 2026 Sep
Volume: 14
Issue: 9
Electronic Location ID: e70525
Copyright: © 2026 The Authors. Immunity, Inflammation and Disease published by John Wiley & Sons Ltd.
License: This is an open access article under the terms of the http://creativecommons.org/licenses/by/4.0/ License, which permits use, distribution and reproduction in any medium, provided the original work is properly cited.
License URL: https://creativecommons.org/licenses/by/4.0/

Figure count: 0
Table count: 0
Page count: 3

source-schema-version-number: 2.0
cover-date: September 2026
details-of-publishers-convertor: Converter:WILEY_ML3GV2_TO_JATSPMC version:6.8.7 mode:remove_FC converted:07.09.2026

==============================

Volume 14, Number 9

September 2026

### Immunity, Inflammation and Disease

Editor-in-Chief
Alexander Hutchison

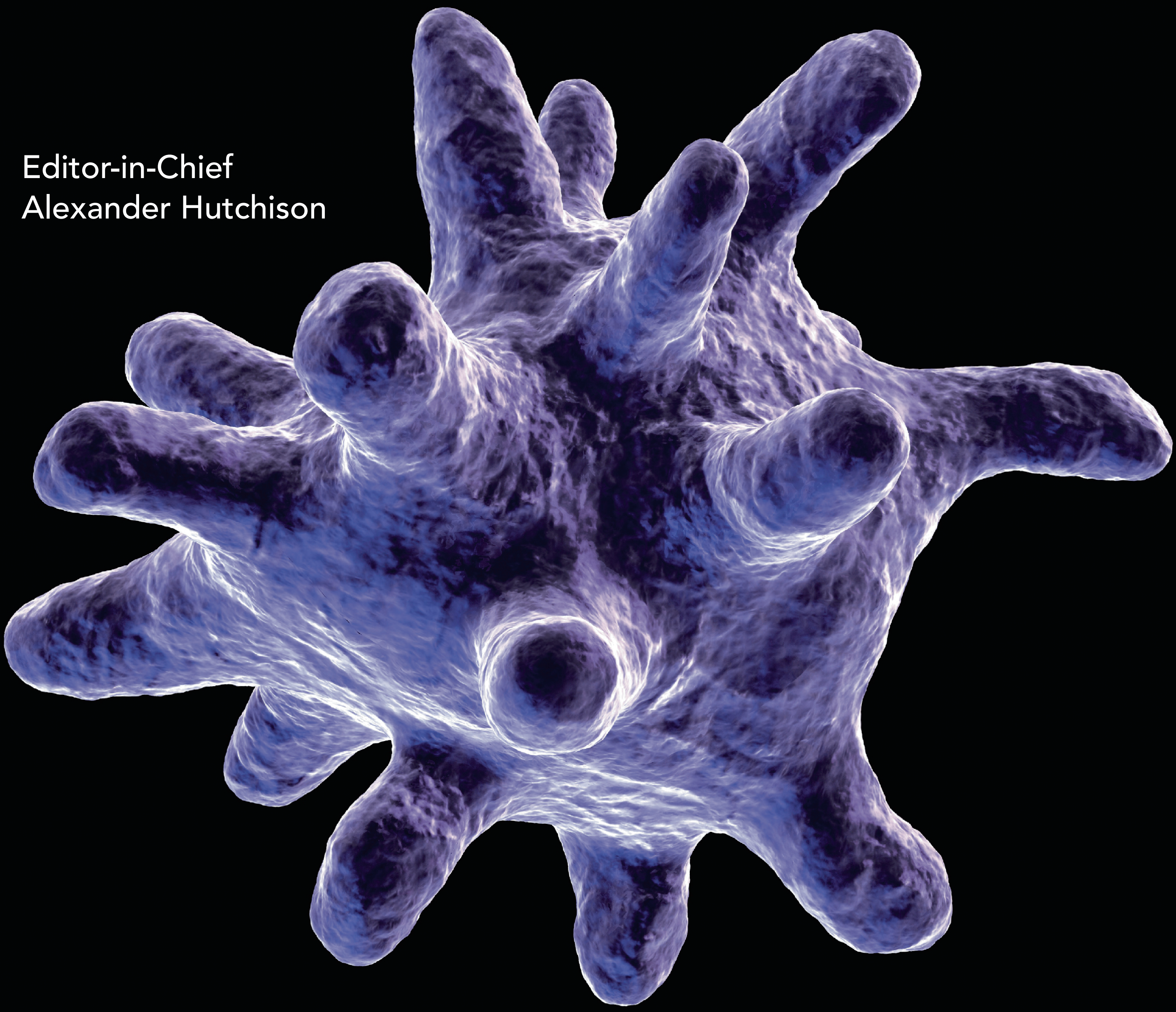

WILEY

Forward
Series

### Immunity, Inflammation and Disease

Open Access

### Aims and Scope

*Immunity, Inflammation and Disease* is a peer-reviewed, open access, interdisciplinary journal providing rapid publication of cutting-edge research across the broad field of immunology. *Immunity, Inflammation and Disease* gives rapid consideration to papers in all areas of clinical and basic research. The journal welcomes original work that enhances the understanding of immunology in areas including cellular and molecular immunology, clinical immunology, allergy, immunochemistry, immunogenetics, cancer immunology, and many others.

Original research articles, methods papers, editorials and commentaries are published. Original research papers must report well-conducted research with conclusions supported by the data presented in the paper. *Immunity, Inflammation and Disease* considers for publication both papers submitted directly to the journal and those referred from a select group of prestigious journals published by Wiley Blackwell. For further information, please refer to our Author Guidelines available at http://onlinelibrary.wiley.com/journal/10.1002/(ISSN)2050-4527/homepage/ForAuthors.html

### Open Access and Copyright

*Immunity, Inflammation and Disease* is a Wiley Open Access journal, one of a series of peer-reviewed titles publishing quality research with speed and efficiency. For further information, please visit the Wiley Open Access website at http://www.wileyopenaccess.com/view/index.html

All articles published by *Immunity, Inflammation and Disease* are fully open access: immediately freely available to read, download and share. Articles are published under the terms of the Creative Commons Attribution License which permits use, distribution and reproduction in any medium, provided the original work is properly cited.

Copyright on any research article in a journal published by *Immunity, Inflammation and Disease* is retained by the author(s). Authors grant Wiley a licence to publish the article and identify itself as the original publisher. Authors also grant any third party the right to use the article freely as long as its integrity is maintained and its original authors, citation details and publisher are identified. Further information about open access license and copyright can be found in this website: http://www.wileyopenaccess.com/details/content/12f25db4c87/Copyright--License.html

### Disclaimer

The Publisher and Editors cannot be held responsible for errors or any consequences arising from the use of information contained in this journal; the views and opinions expressed do not necessarily reflect those of the Publisher and Editors, neither does the publication of advertisements constitute any endorsements by the Publisher and Editors of the products advertised.

Wiley Open Access articles posted to repositories or websites are without warranty from Wiley of any kind, either express or implied, including, but not limited to, warranties of merchantability, fitness for a particular purpose, or non-infringement. To the fullest extent permitted by law Wiley disclaims all liability for any loss or damage arising out of, or in connection with, the use of or inability to use the content.

### Editor-in-Chief

Alexander Hutchison

*Associate Director, Editorial, Wiley*
*Email: Alexander Hutchison (ahutchison@wiley.com)*

*Address correspondence to the Editorial Office:*
*Email: IIDeditorial@wiley.com*

### Editorial Board

Sakib Al Hasan, MD
*Department of Pharmacy, Gopalganj Science and Technology University (GSTU), Gopalganj, Bangladesh*

Heather L. Caslin, PhD
*University of Houston, Houston, TX, USA*

Niels Olsen Saraiva Câmara, PhD
*Institute of Biomedical Science, University of São Paulo, Sao Paulo, Brazil*

Yuezhou Chen, MD, PhD
*Peking University, Peking, China*

Debjani Dutta
*National Institute of Technology, Durgapur, India*

Dorota Formanowicz, MD, PhD
*Poznań University of Medical Sciences, Poznań, Poland*

Alessandro Granito, PhD
*Universitá di Bologna, Bologna, Italy*

Jinming Han, MD, PhD
*Xuanwu Hospital, Capital Medical University, Beijing, China*

Kerry Hilligan
*Malaghan Institute of Medical Research, Wellington, NZ*

Emil Johansson, PhD
*Lund University, Lund, Sweden*

Rajendra Karki, PhD
*Seoul National University, Seoul, South Korea*

Rekha Khandia, PhD
*Barkatullah University, Madhya Pradesh, India*

Feriha Fatima Khidri, MBBS, PhD
*Liaquat University of Medical Health Sciences, Jamshoro, Sindh, Pakistan*

Olanrewaju Morenikeji, PhD
*Virginia State University, Ettrick, VA, USA*

Shreya Mukhopadhyay, PhD
*Merck, New Jersey, United States*

Kim M. O'Sullivan, PhD
*Monash University, Melbourne, Victoria, Australia*

Gabriel A. Rabinovich, PhD
*University of Buenos Aires, Buenos Aires, Argentina*

Aravind Reddy, PhD
*VA Pittsburgh Healthcare System Pittsburgh, PA, USA*

Ritis Kumar Shyanti, PhD
*Alabama State University, Montgomery, AL, USA*

Matthew Sinton, PhD
*University of Manchester, Manchester, UK*

Mukesh K. Sriwastva, PhD
*University of Louisville, Louisville, KY, USA*

Alexis Stamatikos, PhD
*Clemson University, SC, United States*

Kazuo Sugimoto, MD, PhD
*Beijing University of Chinese Medicine, Beijing, China*

Geetika Verma, PhD
*University of North Dakota, Grand Forks, ND, USA*

Jing Wang, PhD
*Shanghai Institute of Immunology, Shanghai, China*

Liang Wang, PhD
*Guangdong Provincial People's Hospital, Guangdong, China*

### Emerging/Reviewing Editors

Nashrah Ahmad, PhD
*Postdoctoral associate*
*Law lab*
*Scripps Research*
*La Jolla, CA, USA*

Tang Bufu, PhD
*Zhongshan Hospital*
*Fudan University, Department of Radiation Oncology*
*Shanghai, China*

Noha Mousaad Elemam, PhD
*Post-Doctoral Research Associate*
*Sharjah Institute for Medical Research*
*College of Medicine, University of Sharjah*
*Sharjah, U.A.E.*

Ninoshka R.J. Fernandes, PhD
*Senior Scientist*
*AbbVie*
*Worcester MA, USA*

Jinming Han, MD, PhD
*Neuroimmunology Center, Department of Neurology*
*Xuanwu Hospital,*
*Capital Medical University, Bejing*
*National Center for Neurological Disorders, China*

Feriha Fatima Khidri, MBBS, PhD
*Associate Professor*
*Liaquat University of Medical and Health Sciences, Jamshoro, Pakistan*

Ravi Shankar Singh, PhD
*Postdoctoral Research Associate*
*Laboratory of Innate Immunity and Signal Transduction,*
*Department of Pathology, Division of Microbiology and Immunology,*
*University of Utah School of Medicine,*
*Salt Lake City, UT, USA*

Goutam Kumar Tanti, PhD
*Postdoctoral Scientist*
*Klinikum Rechts der Isar*
*Munich, Germany*

Geetika Verma, PhD
*Postdoctoral Researcher*
*Deptartment of Biomedical Sciences*
*University of North Dakota School of Medicine & Health Sciences*
*Grand Forks, ND, USA*

Liang Wang, PhD
*Principal Investigator*
*Guangdong Provincial People's Hospital*
*Guangzhou, Guangdong Province*
*China*
